# Successful salvage of a severe COVID-19 patient previously with lung cancer and radiation pneumonitis by mesenchymal stem cells: a case report and literature review

**DOI:** 10.3389/fimmu.2024.1321236

**Published:** 2024-02-06

**Authors:** Xiaohua Huang, Xin Tan, Xiuwen Xie, Tingshu Jiang, Yang Xiao, Zenghui Liu

**Affiliations:** ^1^ Department of Hematology, The First Affiliated Hospital of Guangzhou University of Chinese Medicine, Guangzhou, China; ^2^ Department of Hematology, Dongzhimen Hospital of Beijing University of Chinese Medicine, Beijing, China; ^3^ Department of Rehabilitation Medicine, Southern Theater General Hospital, Guangzhou, China; ^4^ The First Clinical Medical College, Guangzhou University of Chinese Medicine, Guangzhou, China; ^5^ Department of Respiratory and Critical Care Medicine, Yantai Yuhuangding Hospital, Yantai, China; ^6^ Department of Hematology, Shenzhen Qianhai Shekou Pilot Free Trade Zone Hospital, Shenzhen, China

**Keywords:** severe pneumonia, COVID-19, mesenchymal stem cells, lung cancer, immune modulation, tissue repairment

## Abstract

During the COVID-19 pandemic, elderly patients with underlying condition, such as tumors, had poor prognoses after progressing to severe pneumonia and often had poor response to standard treatment. Mesenchymal stem cells (MSCs) may be a promising treatment for patients with severe pneumonia, but MSCs are rarely used for patients with carcinoma. Here, we reported a 67-year-old female patient with lung adenocarcinoma who underwent osimertinib and radiotherapy and suffered from radiation pneumonitis. Unfortunately, she contracted COVID-19 and that rapidly progressed to severe pneumonia. She responded poorly to frontline treatment and was in danger. Subsequently, she received a salvage treatment with four doses of MSCs, and her symptoms surprisingly improved quickly. After a lung CT scan that presented with a significantly improved infection, she was discharged eventually. Her primary disease was stable after 6 months of follow-up, and no tumor recurrence or progression was observed. MSCs may be an effective treatment for hyperactive inflammation due to their ability related to immunomodulation and tissue repair. Our case suggests a potential value of MSCs for severe pneumonia that is unresponsive to conventional therapy after a COVID-19 infection. However, unless the situation is urgent, it needs to be considered with caution for patients with tumors. The safety in tumor patients still needs to be observed.

## 1 Introduction

Since the winter of 2019, the pandemic of coronavirus disease 2019 (COVID-19), caused by severe acute respiratory syndrome coronavirus 2 (SARS-CoV-2), has threatened global public health. COVID-19 is a self-limiting disease for most people. However, approximately 22.97% of cases of COVID-19 developed into acute respiratory distress syndrome (ARDS), and 9.68% of patients needed admission to intensive care unit, with a mortality rate of 1.28% in 2020 (1). The virulence of SARS-CoV-2 has significantly weakened nowadays (2). However, people with old age or frail condition still face a high risk of progression to severe or critical disease of COVID-19. The risk factors associated with severe disease include age more than 60 years old, smoking, being unvaccinated against COVID-19, HIV infection, and underlying noncommunicable illness (1, 3). Any kind of malignancy history was an independent risk factor for severe illness (4) and mortality (5). The estimated pooled mortality rate was 5.6% for the whole population. Nevertheless, it significantly increased to 22.4% for all malignancies, and 32.9% for lung cancer (6). Therefore, lung cancer patients were at a greater risk of death than other types of carcinoma in the pandemic of COVID-19 (7).

As a self-limiting disease, supportive care is essential for all patients (3). Furthermore, the frontline drugs strongly recommended by WHO are nirmatrelvir/ritonavir for non-severe disease and corticosteroids, interleukin-6 (IL-6) receptor blockers, and baricitinib for severe or critical illness (8). The conditional treatments include ruxolitinib, tofacitinib, convalescent plasma, molnupiravir, remdesivir, and so on (8). Nirmatrelvir/ritonavir can significantly reduce the 30-day risk of hospitalization or death by approximately 11.16 per 1000 patients. There are patients (approximately 2.30%) who did not respond to this drug, which include 0.25% who needed ICU admission, 0.083% under mechanical ventilation, and 0.125% who died (9). It is believed that tissue damage co-exists with cytokine storms caused by hyper-activating the immunity system in severe and critical disease (10). On the other hand, baricitinib and tocilizumab, two widely used inflammatory factor inhibitors, also improve the survival of patients with severe or critical COVID-19. However, approximately 18.0% of patients treated with baricitinib and 24% of patients treated with tocilizumab did not respond to these drugs, among whom 56.0% and 46.7%, respectively, progressed to death (11, 12). Thus, the therapy that possesses the ability of inflammatory modulation and tissue repair might be the ideal choice for those who do not respond to frontline treatment. Mesenchymal stem cells (MSCs) have been reported to possess multi-potency (13), which leads to MSCs being widely used in regenerative medicine and tissue engineering. Nowadays, MSCs’ ability related to immunomodulation is proven as well (14). Therefore, MSCs can theoretically be used to treat severe and critical COVID-19 patients who do not respond to frontline drugs, and the efficacy has been proven by many clinical trials (15–35). However, MSCs are rarely used in cancer patients due to concerns about safety and neoplasm recurrence. Hence, the safety and efficacy of MSCs for cancer patients are still unclear. Here, we report a patient with lung cancer. She suffered from severe pneumonia caused by COVID-19 following radiation pneumonitis and was finally successfully salvaged by treatment with MSCs after the failure of the first-line treatment, which brings in hopes of offering clinicians evidence and confidence in MSCs utilization.

## 2 Case presentation

### 2.1 Recent medical history

A 67-year-old female patient had suffered from a recurring pain in the neck, shoulder, and left upper limb for more than 3 months since August 2021, so she visited the Yantai Yuhuangding Hospital on November 26, 2021. The laboratory tests indicated that carcinoembryonic antigen (CEA) was at 34.5 ng/mL and neuron-specific enolase (NSE) was at 19.1 ng/mL, and a BI-RADS 4a focal and bilateral axilla lymphadenectasis were detected by color ultrasonic scan. The CT scan suggested a large focal in the left lung superior lobe. No evidence proved the existence of an infectious disease, and therefore she was hospitalized and went through a CT-guided percutaneous core needle biopsy of the lung on December 2, 2021. The pathological and molecular biological results supported the diagnosis of non-small cell lung cancer (adenocarcinoma) with epidermal growth factor receptor (EGFR) and TP53 mutation. She was finally diagnosed with lung adenocarcinoma of the left superior lobe (cT3NxM1c, with hilar, mediastinum, neck vertebra, and lumbar vertebra metastasis and EGFR and TP53 mutation). She visited the oncology department and received oral EGFR inhibitor osimertinib based on the doctor’s recommendations and the National Comprehensive Cancer Network (NCCN) guidelines. The periodic lung CT examination after oral osimertinib showed a significant reduction of lung lesions. Then, she underwent irradiation therapy from June to August 2022. In November, she began coughing, with phlegm, and later developed shortness of breath, with no fever, and had chest pain, and she was hospitalized in Yantaishan Hospital. She was diagnosed with tuberculosis, aspergillus, and pneumocystis jirovecii infection and treated with isoniazid, pyrazinamide, ethambutol, quinolone antibiotics, sulfamethoxazole/trimethoprim, voriconazole, and glucocorticoid. The symptoms were significantly alleviated after the anti-infective therapy, but she still had cough and phlegm sometimes. The respiratory symptoms were exacerbated again on December 18, 2022, so the patient visited the respiratory department of Yantai Yuhuangding Hospital on December 19, 2022 and was hospitalized. She felt dyspnea and had no fever at admission. The test for SARS-CoV-2 RNA performed 1 day before hospitalization yielded a negative result.

### 2.2 Medical history

The patient had a history of hypertension stage 2 and coronary atherosclerotic cardiopathy (CAD) for approximately 20 years, but recently, her blood pressure was normal and she had no sign of CAD without any intervention.

### 2.3 SARS-CoV-2 vaccination history

The patient was vaccinated with a first dose of inactivated anti-SARS-CoV-2 vaccine (Vero cell) on May 20, 2021, and the second dose was received on June 17, 2021.

### 2.4 Physical examination

At admission, her body temperature was 36.7°C, her pulse rate was 97/min, her respiratory rate was 23/min, and her blood pressure was 146/79 mmHg. SpO_2_ was 99% with a 3 L/min of nasal oxygen supply. Other physical examinations revealed no specific signs, but the Velcro rale was auscultated.

### 2.5 Laboratory test at hospitalization

The emergency laboratory test results were as follows: blood routine—white blood cell count (WBC), 8.02 × 10^9^/L; neutrophil count (NEU), 7.53 × 10^9^/L; hemoglobin (HGB), 99 g/L; platelet (PLT), 176 × 10^9^/L; lymphocyte count (LYM), 0.22 × 10^9^/L; D-dimer (DDi), 2.24 mg/L; procalcitonin (PCT), 0.0916 ng/mL; and high sensitivity C-reactive protein (hs-CRP), 42.65 mg/L. ProBNP, cardiac biomarkers, hepatic and kidney function tests, and electrolyte panel were not significantly abnormal.

### 2.6 Therapeutic intervention and outcome

#### 2.6.1 Frontline treatment and outcome

**Figure 1:**
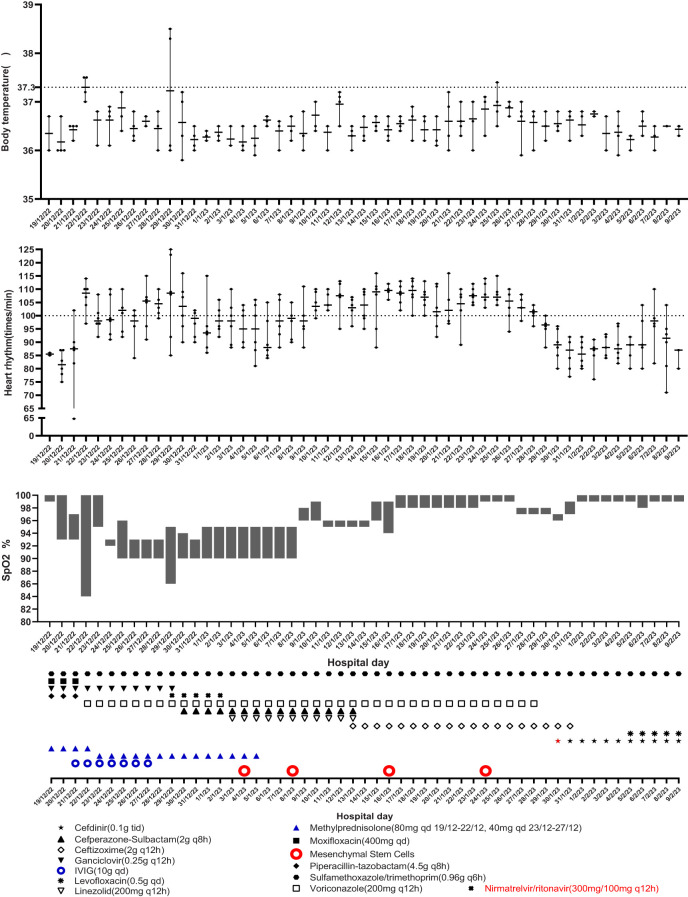
Symptom and treatment.

Given the patient’s poor lung condition caused by radiation therapy and suppressed immunity caused by anti-tumor agents, the patient was at a high risk of opportunistic infection. Sulfamethoxazole/trimethoprim (SMZ/TMP, 0.96 g q6h), moxifloxacin (400 mg qd), piperacillin-tazobactam (4.5 g q8h), ganciclovir (0.25 g q12h), methylprednisolone (80 mg qd), and supportive therapy were empirically administrated on the first day. However, the patient’s symptoms were not significantly alleviated after 2 days of treatment. The heart rate accelerated and SpO_2_ were decreased, which meant a more serious anoxia than before. The test results for SARS-CoV-2 RNA (swab), (1,3)-*β*-D-glucan (peripheral blood), and galactomannan (peripheral blood) were negative. A chest X-ray tomography was arranged on December 22, 2022, and it indicated a multiple ground-glass shadow of the lung. Considering the poor immunity result from a previous carcinoma history, intravenous immunoglobulin (10 g qd) was used from December 21 to 27, 2022. Moxifloxacin and piperacillin–tazobactam were ceased, and voriconazole (200 mg q12h) was added by December 22, 2022, given her history of aspergillus and high risk of aspergillus re-infection, and we reduced methylprednisolone as well (40 mg qd) (**Figure 1**).

**Figure 2:**
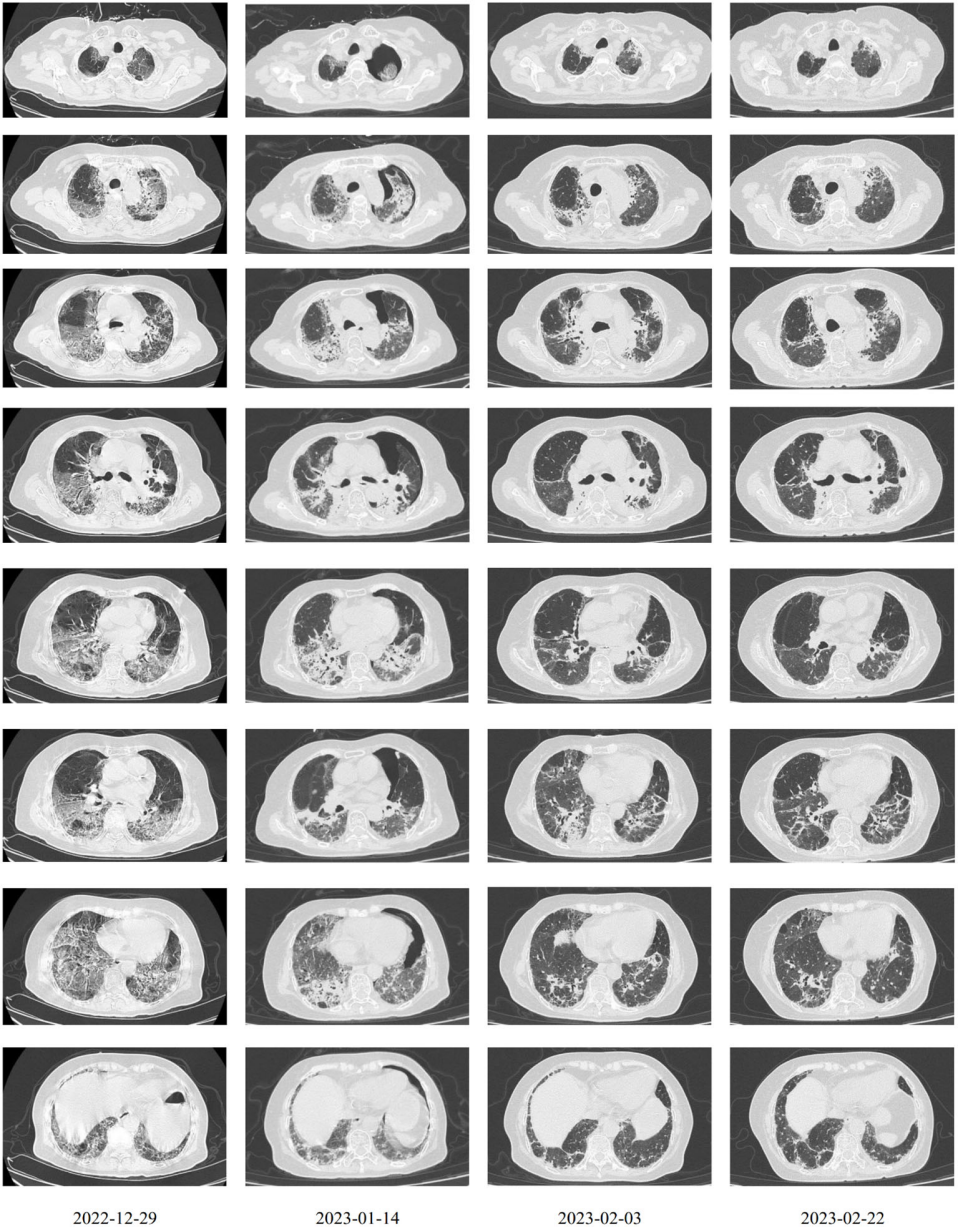
Characteristics of CT graphs.

After observing the abovementioned treatment for another week, the patient’s condition was still unsatisfactory. Although the body temperature and blood pressure were normal, the patient often coughed with phlegm, had dyspnea, and was fatigued. The SpO_2_ fluctuated between 84% and 94% with nasal oxygen supply. On December 29, 2022 the COVID-19 RNA test was positive. Chest computerized tomography suggested new bilateral interstitial pneumonia (**Figure 2**). Ganciclovir was ceased, and paxlovid was administrated from December 29, 2022. Cefoperazone–sulbactam (3 g q8h) was also added starting December 30, 2022.

However, no alleviation of the patient’s symptoms was observed after the 5-day treatment including nirmatrelvir/ritonavir. Although we tried the addition of linezolid (200 mg q12h) since January 3, 2023, the symptoms were still serious. Therefore, we advised the utilization of MSCs for salvage therapy.

#### 2.6.2 Salvage treatment by MSCs

##### 2.6.2.1 MSCs preparation and ethical consideration

Clinical-grade human umbilical cord mesenchymal stem cells (hUC-MSCs) were supplied by the umbilical cord blood bank of Shandong Province. The preparation was completed in a GMP laboratory. Samples of human umbilical cords were collected and cut into 2- to 3-mm pieces to be processed for MSCs isolation. Small cylindrical fragments (*d* = 2 mm) were removed from the mucous connective tissue (Wharton’s jelly matrix), avoiding blood vessels and amniotic epithelium. They were transferred to culture dishes without any blood serum for adherent culture. Wharton’s jelly fragments were incubated in human MSCs growth medium at 37°C in a humidified incubator under a 21% O_2_ and 5% CO_2_ atmosphere. After 7–10 days of culture, the first colonies of WJ-MSCs were observed. Then, the cells were pursued until subconfluence; the non-adherent cells were removed, and the stromal cells were detached (0.05% trypsin-ethylenediaminetetraacetic acid (trypsin-EDTA)) and then transferred into 25-cm^3^ flasks at an initial density of 5 × 10^3^/cm^3^ and cultured up to 70%–80% confluence before collection for subsequent passages. Cells at passages P5 were used and had the International Society for Cell & Gene Therapy (ISCT)-recommended cell surface characteristics of MSCs, including expression (95%) of clusters of differentiation 73 (CD73), CD90, and CD105 and lack of cell surface presentation (<2%) of CD34, CD45, CD14 or CD11b, CD79*α* or CD19, and human leukocyte antigen-DR (HLA-DR).

Intravenous administration was used. Before the intravenous drip, the hUC-MSCs were suspended in 100 mL of normal saline, and the total number of transplanted cells was calculated as 1 × 10^6^ cells/kg. The MSCs were infused through the patient’s right cubital veins for approximately 1 h (35 drops/min).

It had received approval from the Ethics Committee of Yantai Yuhuangding Hospital for compassionate use. Informed consent was obtained from the patient and her family members.

##### 2.6.2.2 Efficacy of MSCs

**Figure 3:**
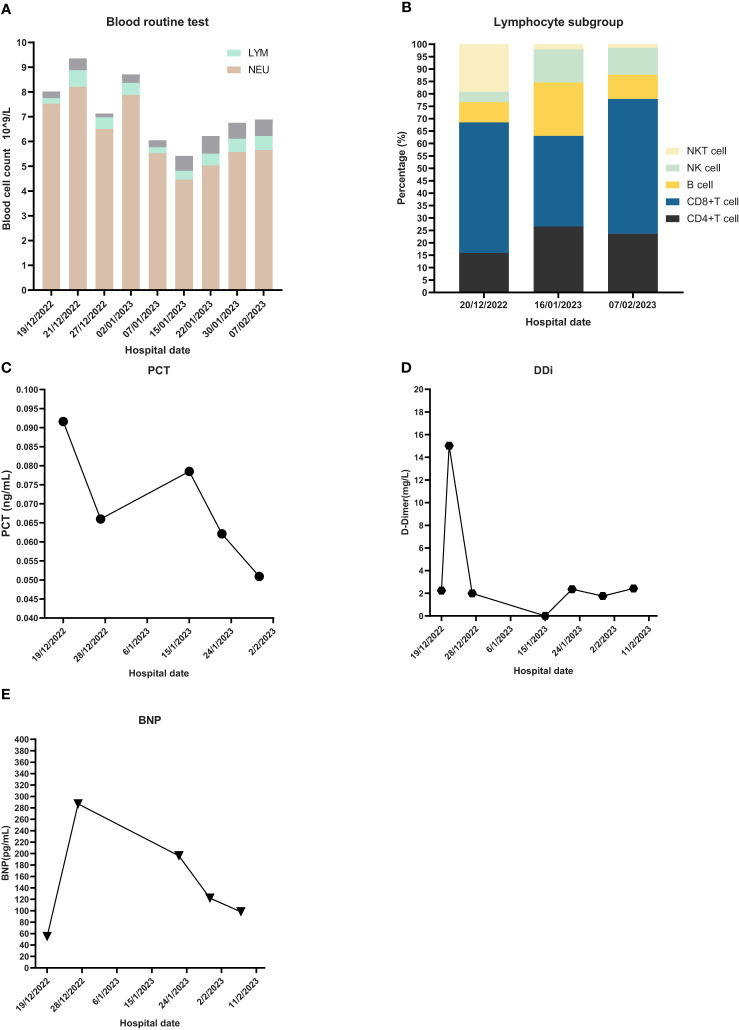
Laboratory Inspection. **(A)** Blood routine test and proportion of different blood cells. NEU: neutrophil, LYM: lymphocyte. **(B)** Lymphocyte subgroup analysis. **(C)** PCT: Procalcitonin. **(D)** DDi: D-Dimer. **(E)** BNP: B-type natriuretic peptide.

The first dose (4.93 × 10^7^ cells) of MSCs was intravenously infused on January 4, 2023. Her difficulty in breathing was slightly alleviated. The second dose (4.88 × 10^7^ cells) was infused on January 8, which led to an advancing improvement. SpO_2_ was higher than before and fluctuated between 95% and 99% after the second dose of MSCs. However, on January 14, 2023, the chest CT indicated a slightly extending bilateral pneumonia and novel pneumothorax of the left lung. Serum interleukin-6 (IL-6) was 34.17 pg/mL (**Figure 3**), which was believed to be relative to severe COVID-19 (36). PCT was 0.0785 ng/mL and hs-CRP was 9.68 mg/L. We were concerned that the patient’s condition would be exacerbated again, so the third dose (4.94 × 10^7^ cells) of MSCs was arranged in the morning of January 16, 2023. Although the patient had six times of hemoptysis at night, it was quickly controlled the next day and the condition became much better 3 days later. Cefoperazone–sulbactam was changed into ceftizoxime (2 g q12h) in January 14 as well because of limited evidence of serious bacterial infection (low level of PCT detected and normal count of WBC and NEU on weekly surveillance). The fourth and last dose (4.95 × 10^7^ cells) of MSCs for consolidation therapy was administrated on January 24, 2023. The patient no longer relied on middle to high oxygen concentration since January 25, 2023. A lung X-ray suggested an improvement of right pneumonia and absorption of the left pneumothorax. All symptoms were alleviated significantly, and her performance status recovered. We prescribed oral antibiotics since February 2, 2023, and the patient was eventually discharged 2 days later. The patient is still alive since the last follow-up on July 15, 2023.

## 3 Discussion

We report a patient with a poor lung condition due to carcinoma and radiotherapy. Complex infections, including bacteria, fungus, and COVID-19, further impaired the lung. Nevertheless, bacteria and fungus might not be the vital pathogen in this case because of continuously low levels of PCT, WBC, NEU, and (1,3)-*β*-D-glucan and the characteristics of chest CT. The utilization of broad-spectrum antibiotics and antifungal agents did not effectively reverse the pneumonia. The patient received nirmatrelvir/ritonavir immediately when the SARS-CoV-2 RNA test was positive. It was suggested that the hospital admission or death rate of patients who were given nirmatrelvir/ritonavir was only 2.1% (37). However, she developed a severe disease in a short time. The mortality of all lung cancer patients with COVID-19 was 31.0% in a multi-country pooled analysis (38). It increased to 58.4% when patients developed severe disease (39). Therefore, lung cancer patients are at higher risk of death, and the risk would incredibly increase when patients progress to severe COVID-19. We believed that the patient’s condition was less likely to alleviate without advance treatment, and it was urgent and necessary at that time to find a suitable treatment as soon as possible to reverse the disease quickly. The lung injury due to inflammation and former radiation pneumonitis (RP) were a concern, and she needed a treatment option that had a function of immunomodulation and injury repair. The frontline inflammatory inhibitor baricitinib (JAK inhibitor) and tocilizumab (IL-6 inhibitor) could be choices, but they were both hard to acquire at that time and had no evidence of tissue repair effect. In addition, remdesivir is also an option. However, the Chinese Food and Drug Administration did not approve the marketing of remdesivir in China, which may be because, according to the results of several randomized controlled trials, Chinese patients may not benefit from the drug. These trials showed that there was no significant difference in the recovery rate and the risk of progression to severe disease between remdesivir and the control group (40–42). The patient received four doses of MSCs infusion and finally achieved a surprisingly effective outcome. The case indicates that patients with a poor condition of the lung and serious inflammation may benefit from MSCs.

### 3.1 Potential mechanism of MSCs

ARDS or acute lung injury caused by SARS-CoV-2 induces cytokine storm characterized by severe hypoxemia, high-permeability pulmonary edema, and reduced compliance of the integrated respiratory system as a result of widespread compressive atelectasis and fluid-filled alveoli (43). Moreover, the virus triggers alveolar and interstitial fibrin deposition, endothelial dysfunction, and pulmonary intravascular coagulation, which may also contribute to the persistence of abnormal inflammatory and disease progress. Recent studies revealed that the biological mechanism of MSCs for the treatment of infectious lung injury might involve multiple pathways. However, immunomodulation and tissue repair might be both crucial.

#### 3.1.1 Immunomodulation

SARS-CoV-2 enters cells, and cells’ capillary permeability increases, allowing more viruses to transfer into cells, resulting in ARDS. The virus activates an immune response involving natural killer (NK) cells, CD4^+^T cells, CD8^+^T cells, and B cells in COVID-19 patients, causing the abnormal production of cytokines (44). The abnormal cytokine profile results in excessively activated but dysfunctional immunity reactions in patients with severe COVID-19, which presents a delayed or failed elimination of virus. It has been proven that MSCs affect both innate and acquired immunity to modulate the abnormal immune reaction in patients.

Induction of inflammatory cytokines from macrophages by the nucleocapsid (N) protein of SARS-CoV-2 has been proven. N-protein could promote tumor necrosis factor (TNF), interleukin-1*β* (IL-1*β*), interleukin-6 (IL-6), and monocyte chemoattractant protein-1 (MCP-1) secretion of macrophages, which was associated with the occurrence of severe COVID-19 (45). MSCs affect macrophages through contact-dependent interaction, paracrine-mediated mechanisms, autophagy, mitophagy, and oxidative stress (46). With MSCs’ existence, pro-inflammatory macrophages secrete anti-inflammatory factors and pro-inflammatory cytokines were downregulated, consequently resulting in the enhancement of phagocytic properties and macrophage type switch (47). In an experiment in mice, a significant reduction in inflammatory cell and alveolar hemorrhage were observed, and the total number of macrophages and neutrophils, respectively, were remarkably decreased (48).

In patients with severe COVID-19, NK cell was activated by activating ligands or pro-inflammatory cytokines produced by NK cell itself (49) or other cells. However, activating receptors on NK cells and transcription factor T-bet were both downregulated, and transforming growth factor-*β* (TGF*β*) inhibited the function of NK cells. This resulted in NK cell exhaustion, impairment, disability to degranulate, and cytotoxicity (50). The immunological profile in patients with COVID-19 is characterized by the contraction of immature CD56^bright^ and expansion of mature CD57^+^Fc*ϵ*Rlγ^neg^ adaptive NK cells, which might lead to hyperactive inflammation (51). Several studies have proven that CD107*α* expression of NK cells was observed when NK cells were co-cultured with MSCs, which suggested that MSCs could effectively assist in NK cell degranulation (52).

Dendritic cells (DCs) are major antigen-presenting cells of innate immunity and bridge adaptive immunity. SARS-CoV-2 promotes the expression of major histocompatibility complex (MHC) molecules and co-stimulatory receptors of DCs that represent its maturation. Additionally, the nuclear factor-*κ*B (NF-*κ*B) pathway, which is believed to be vital to the signal responsible for the expression and secretion of pro-inflammatory cytokines, is activated when DCs interact with virus protein (53, 54). MSCs have the capacity to inhibit DC maturity and induce mature DC (mDC) differentiation into regulatory DC by many pathways involving notch-1/Jagged-1 signal (55–57), Akt signal (58), C-C motif chemokine receptor 7 (CCR7) gene degradation (59), T helper 17 (Th17) cell inhibition (60), and so on.

At the aspect of T cell, the virus-specified T cell and bystander T cell are both activated in the early stage of infection (61). However, the unresolved inflammation due to high exposure dose or rapid replication of virus, age, and deficient immunity may result in prolonging the activation of bystander T cells, which can produce pro-inflammatory cytokines continuously (62). Those long-lasting T cells in severe COVID-19 are dysfunctional and pathological, and massive activation-induced cell death is observed (63, 64). MSCs demonstrate an immunomodulatory effect on CD4^+^ T cells’ subgroup balance and an immunosuppressive effect on CD3^+^ T cells. MSCs inhibited Th17 and induced Treg differentiation, which led to the attenuation of lung injury caused by the virus (65). On the other hand, when MSCs were injected in mice with viral pneumonia, attenuated proliferation of both CD3^+^, CD4^+^ and CD4^+^, CD8^+^ T cells was observed, resulting in the significant suppression of pro-inflammation factor level and the protection of mice alveolar epithelial cells from inflammatory injury similar to severe COVID-19 (66).

The antibody against SARS-CoV-2 is secreted by B cells. In patients with a severe disease, an impaired germinal center response and a robust extrafollicular response were observed compared to those with a mild infection, which might be associated with an elevated level of antibody (perhaps low-mutation IgG1) and pro-inflammatory cytokines. The abnormal activation of extrafollicular B cells in severe COVID-19 is similar to autoimmune response and correlated to an increasing level of inflammation hallmarks and death (67–69). When co-cultured with MSCs, most proteins of the pathway activating B cells were significantly reduced, resulting in the reduction of cell proliferation and transcription gene expression involving terminal differentiation into plasma cell of B cell (70). Regulatory B cell (Breg) is a new subgroup recently defined with a function similar to Treg, and Breg is increased by MSCs. IL-10 secreted by Breg was increased, and immunoglobulin production was further inhibited (71). MSCs were believed to ameliorate lung tissue injury by inhibition of chemotaxis gene and immunoglobulin expression at the aspect of humoral immunity (72).

In summary, MSCs are believed to be effective for severe COVID-19 by the immunity pathways of reducing pro-inflammatory factor through multiple signal pathways, consequently promoting macrophage phenotype switch, NK cell degranulation, regulatory DC generation, Treg differentiation, and B cell activation inhibition.

#### 3.1.2 Tissue repair

There are two types of lung damage relative to severe COVID-19: one induced by inflammation, which possibly induces ARDS and the other connected with fibrosis, also known as post-COVID pulmonary fibrosis. At first, in the acute phase, the virus causes diffuse and severe alveolar damage, including critical endothelial injury, microangiopathy, and obstruction of the alveolar capillaries. After this, the pulmonary surfactant becomes dysfunctional and deficient, contributing to edema and fibrosis. In severe cases, the abovementioned process is continuous and transforms normal lung tissue into fibrous tissue eventually (73).

MSCs demonstrate multi-potency and high proliferation ability in many studies, which make MSCs an ideal option for regenerative medicine. The extracellular vesicles (EVs) from MSCs are released and contact with the target recipient cell. Afterward, the damaged lung tissue is protected from inflammatory injury and renewed (74, 75). When MSCs were co-cultured with injured lung tissue *in vitro*, 44 kinds of proteins were secreted to accelerate airway epithelium repair by stimulating migration, proliferation, and differentiation. MSCs enhanced anti-apoptosis signal pathways, restored matrix metalloproteinases function, and reduced the percentage of type II alveolar epithelial cells (76–78). An analysis of EVs’ miRNA indicated that miR-223-3p, which is associated with the alleviation of pulmonary fibrosis, was abundant in EVs, and it could attenuate the deposition of fibrosis-related factors. *In vitro*, EVs restricted the activation and proliferation of fibroblasts (79). Additionally, miR-214-3p and miR-466f-3p derived from MSCs mediated prevention from radiation injury and inhibited lung fibrosis by downregulating the ATM/P53/P21 signaling and inhibiting the AKT/GSK3*β* pathway, respectively (80, 81).

Therefore, MSCs repair injured lung tissue caused by severe COVID-19 disease probably through promoting molecules involved in airway epithelium proliferation and differentiation, activation of anti-apoptosis signal pathways, and inhibition of fibroblasts.

### 3.2 Clinical use and research

#### 3.2.1 Case reports

MSCs have been tried as a salvage treatment for critically ill pneumonia patients after the outbreak of the pandemic. In 2020, Chinese physicians successively shared several cases of severe pneumonia treated with MSCs (82–88), and then similar cases began to be reported in other countries and regions (87–96). The dose range for an intravenous drip of MSCs was (1–3) × 10^6^ kg in most of the cases (82–90, 96). It is also important to note that none of these cases had a history of cancer, except for three cases reported in Sweden in 2022 (96) (**Table 1**).

**Table 1:** Characteristics of public case reports of mesenchymal stem cells (MSCs) for patients with severe pneumonia.

Case	Country	Sex	Age	Previous diagnosis	Accompanies	Treatment before MSCs	MSCs type	MSCs administration (cells/time)	Efficacy (from initial MSCs)	Notes
Liang 2020 (82)	China	F	65	Hypertension, Diabetes	MOF, Gastrorrhagia Hemolysis	IFN-α inhalation, Lopinavir/Ritonavir, Moxifloxacin, Xuebijing, Methylprednisolone, Immunoglobulin, Thymosin α1	UC	5×10^7^ VD×3	Remission	Discharged on day 30 (day11 from MSCs)
Peng 2020 (83)	China	F	66	No	No	Convalescent Plasma	UC	1×10^6^/kg VD×3	Remission	Discharged on day 42(day 10 from MSCs)
Soler Rich 2020 (89)	Spain	/	/	No	No	Hydroxychloroquine, Azithromycin, Enoxaparin	BM	1×10^6^/kg VD	Remission	Discharged on day 17 (day 6 from MSCs)
Tang 2020 (84)	China	F	37	No	No	Oseltamivir, Arbidol hydrochloride	MB	1×10^6^/kg VD×3	Remission	
	China	M	71	No	No	Ribavirin, Arbidol hydrochloride, Cefoperazone-Sulbactam	MB	1×10^6^/kg VD×3	Remission	
Tao 2020 (85)	China	M	72	Diabetes, Hypertension, Nephropathy	Renal failure Respiratory failure	Interferon, Lopinavir/Ritonavir, Piperacillin-tazobactam, Methylprednisolone, LMWH, Arbidol, ECMO	UC	1.5×10^6^/kg VD×5 with ECMO	Partial remission	Received lung transplantation on day 37, died on day 43.
Zhang 2020 (86)	China	M	54	Diabetes	No	Lopinavir/Ritonavir, IFN-Α Inhalation, Levofloxacin, Tanreqing capsule, Xuebijing, Thymosin α1, Methylprednisolone, Immunoglobulin Plasma Exchange.	UC	1×10^6^/kg VD	Remission	Discharged from ICU on day 2
Eckard 2020 (87)	America	M	4	No	Bradycardia, Anemia LVEF declined	Immunoglobulins, Steroids, Aspirin, Anti-Coagulants.	BM	2×10^6^/kg VD	Remission	Discharged on day 9
	America	F	10	No	Hypertension	/	BM	2×10^6^/kg VD	Remission	Discharged on day 10
Yilmaz 2020 (88)	Turkey	M	51	No	MOF cardiac arrest brain involvement	Avigan, Tocilizumab	UC	3×10^6^/kg VD+IV×4d With anti-thromboembolic	Remission	Discharged on day 40
Primorac 2021 (90)	Croatia	M	50	No	No	Azithromycin, Methylprednisolone, Amoxicillin/Clavulanic acid, Remdesivir, Enoxaparin, Moxifloxacin, Immunoglobulins	BM	3×10^6^/kg VD With Colistin, Fosfomycin, Linezolid, Ceftazidime/Avibactam.	Remission	Discharged from ICU on day 31, from hospital on day 49
Putra 2021 (91)	Indonesia	M	54	Severe hypertension	cardiomegaly	Remdesivir, Azithromycin, Levofloxacin, Dexamethasone	BM	240×10^6^/L IM×12	Remission	Discharged from ICU on day 23
	Indonesia	M	53	Diabetes	cardiomegaly Lung edema	Oseltamivir, Azithromycin, Levofloxacin,	BM	320×10^6^/L IM×16	Remission	Discharged from ICU on day 19
	Indonesia	M	72	Hypertension, liver failure, stroke sequelae thalassemia	cardiomegaly	Favipiravir, Azithromycin, Tazobactam, Levofloxacin, Dexamethasone	BM	360×10^6^/L IM×18	Remission	Discharged from ICU on day 14
Senegaglia2021 (92)	Brazil	M	51	Hypertension	No	Dexamethasone, Enoxaparin, Tocilizumab	UC	5×10^5^/kg VD×3 With Tocilizumab.	Remission	Discharged from ICU on day 11, from hospital on day 15
Grumet 2022 (93)	America	M	91	No	No	Hydroxychloroquine, Remdesivir, Dexamethasone, Convalescent Serum	PLX-PAD	300×10^6^/L IM×15	Remission	Died of a bacterial infection on day 140
Kim 2022 (94)	Korea	M	73	Diabetes, Hypertension, cerebral infarction, Hyperlipidemia	No	Chloroquine, Lopinavir/Ritonavir, Inotropic agent, Immunoglobulin, Cefepime, Teicoplanin, Steroids, Tracheostomy	BM	90×10^6^/L VD With aspirin	Remission	Discharged on day 138
Payandeh 2022 (95)	Iran	M	28 days	Pneumonia	No	Ampicillin, Cefotaxime, Clindamycin, Meropenem	hPD	7×10^6^/L VD×2	Remission	Discharged on day 20
Sadeghi 2022 (96)	Sweden	M	22	ALL (HSCT), aGVHD, HC, ECMO	MOF	Cyclophosphamide, Fractionated TBI	BM	0.7×10^6^/L VD then 1.4×10^6^/L VD	Remission	Died of MOF on day 104
	Sweden	M	13	ALL (HSCT), Neutropenia	Invasive aspergillosis,		BM	1×10^6^/kg VD	Progression	Died of aspergillus pneumonia
	Sweden	M	33	CML (HSCT), Streptococcal septicemia	No	Cyclophosphamide, Busulfan, Methotrexate, Cyclosporine, Piperacillin/Tazobactam	DSC	1×10^6^/kg VD	Remission	Discharged on day 22 (day11 from MSCs)

DSCs, decidua stromal cells; TBI, total body irradiation; BM, bone marrow; UC, umbilical cord; MB, menstrual blood; LMWH, low molecular weight heparin; ECMO, extracorporeal membrane oxygenation; LVEF, left ventricular ejection fraction; HSCT, hematopoietic stem cell transplantation; MOF, multi-organ failure; PET, pulmonary thromboembolism; aGVHD, acute graft-versus-host disease; HC, hemorrhagic cystitis.

#### 3.2.2 Clinical trials

Many trials have been performed to explore the efficacy of MSCs for patients with severe pneumonia. The common dose of MSCs for intravenous injection was 0.5 to 3 × 10^6^ cell/kg each time for one to three administrations. However, the clinical outcome was varied.

In some small-sample single-arm trials, MSCs demonstrated ideal efficacy with a survival rate of more than 80% (15, 18, 19), while it was just almost 60% in a large-sample single-arm trial (17). A significantly better survival and time to improvement (24) were likewise observed in some cohort studies (23, 24), but MSCs therapy did not dramatically shorten hospital duration (22, 24, 25). In many small-sample randomized controlled trials, MSCs therapy could significantly decrease mortality (27–29, 31), shorten hospital time, and increase the rate of clinical improvement (26, 27, 30, 31, 97). However, the diagnosis of all eligible subjects was COVID-19 pneumonia, without malignancy under treatment.

A long-time follow-up was described in only one trial, but the risk of secondary cancer was not mentioned (97). Limited by a small sample, all other trials also did not explore the risk of secondary malignancy in a long time after MSCs administration (**Table 2**).

**Table 2:** Characteristics of public clinical trials of mesenchymal stem cells (MSCs) for patients with severe pneumonia.

Study	Country	Phase	Type	Diagnosis	Excluded tumor	N1	Treatment group	Dose (10^6^cells)	N2	Control group	Clinical outcome (Treatment vs control)
Sengupta V2020 (15)	America	II	Single-arm	Moderate-to-severe COVID-19	Yes	24	BM-MSC-derived exosomes	15mL qd×14	/	/	Survival rate 83%, 71% recovered and/or discharge, mean hospital duration was 5.6 days after administration.
Hashemian S R 2021 (16)	Iran	I	Single-arm	Severe COVID-19	Malignancy under treatment	11	UC-MSCs (6) or PL-MSCs (5) Standard care	200 qod×3	/	/	5 Survived and 6 died. Patients that develop sepsis or multi-organ failure may not be good candidates for stem cell therapy.
O. Ercelen 2021 (17)	Turkey	I	Single-arm	Severe COVID-19	Yes	222	UC-MSCs Standard care	1-2/kg 1 dose	/	/	138 survive or discharge.
Saleh M 2021 (18)	Iran	I	Single-arm	Severe COVID-19	Yes	5	WJ-MSCs Standard care	150 q3d×3	/	/	4 patients improved in CT assessment.
Chu M 2022 (19)	China	I	Single-arm	Mild to severe COVID-19	No	7	MSC-derived exosomes Standard care	1/kg Bid	/	/	All patients discharge, mean hospital duration was 18.74 days
GrÉGoire 2022 (20)	German	I/II	Single-arm	Severe COVID-19	Yes	8	BM-MSCs Standard care	1.5-3/kg qd×3	/	/	Median hospital duration was 30 days with all discharge. Median ICU duration was 12 days, one need readmission to ICU after MSCs injection.
Zhu Y G 2022 (21)	China	IIa	Single-arm	Severe COVID-19	Yes	7	haMSC-derived exosomes Standard care	200 qd×5	/	/	All patient discharge, and median hospital duration was 26 days.
Meng 2020 (22)	China	I	Cohort study	Moderate-to-severe COVID-19	Yes	9	UC-MSCs Standard care	30 q3d×3	9	Standard care	Hospitalization duration was 20 vs 23 days P=0.306; Mechanical ventilation rate was 11.11 vs 44.44%, P=0.294.
Kaffash F N 2022 (23)	Iran	I	Cohort study	Severe COVID-19	Yes	10	UC-MSCs Standard care	1/kg qod×3	10	Standard care	Mortality rate was 10 vs 20%, P>0.05 Fifth day SPO_2_/FIO_2_ ratio was 151.38 vs 223.83, P=0.003; Tenth day SPO_2_/FIO_2_ ratio was 164.49 vs 248.88, P=0.029.
Xu X 2021 (24)	China	II	Multicenter Cohort study	Severe COVID-19	Yes	44	MB-MSCs Standard care	90 qod×3	18	Standard care	Survival rate was 92.31 vs 12%, P=0.048; Time to improve was 3.0 vs 8.8 days, P=0.049; Hospital duration was 30.65 vs 34.94 days, P=0.413; ICU duration was 24.00 vs 22.17 days, P=0.465.
Aghayan H R 2022 (25)	Iran	I	Multicenter Cohort study	Severe COVID-19	Yes	10	PL-MSCs Standard care	1/kg 1 dose	10	Standard care	28-day survival rate was 50 vs 50%, P>0.05 Duration of hospitalization of recovery patient was almost equal.
Shu L 2020 (26)	China	II	RCT	Severe COVID-19	Yes	12	UC-MSCs Standard care	2/kg	29	Standard care	Clinical improve time was 9 vs 14 days, P=0.006; Day 7 improve rate was 58.33 vs 17.24%, P=0.020 Day 28 mortality was 0 vs 7.31%, P=0.543
Adas G 2021 (27)	Turkey	II	RCT	Severe COVID-19	Yes	10	MSCs Standard care	3/kg qd×3	10	Standard care	Mortality rate was 30 vs 60%, P<0.001; ICU duration was 31 vs 41 days, P<0.05; Hospitalization duration was 47 vs 45 days, P>0.05; Comorbidity rate was not different.
Lanzoni G 2021 (28)	USA	I/IIa	RCT	Severe COVID-19	Yes	12	UC-MSCs Standard care	100 q3d×2	12	Standard care	One month survival was 91 vs 42%, P=0.015 One month recovery was 9 vs 4 pts, P=0.031.
Dilogo I H 2021 (29)	Indonesia	III	RCT	Severe COVID-19	Yes	20	UC-MSCs Standard care	1/kg Once	20	Placebo Standard care	Survival rate was 50 vs 20%, P=0.047; Comorbidities rate was 4.5 vs 1%, P=0.023;
Shi L 2021 (30, 97)	China	II	RCT	Severe COVID-19	Yes	65	UC-MSCs Standard care	40 q3d×3	35	Placebo Standard care	Discharge at day 10 was 16.92 vs 17.14%, P>0.05; Total change in lesion proportion at day 28 was -19.4 vs -7.30%, P=0.08; Total change in solid component lesion proportion at day 28 was -57.7 vs -44.5%, P=0.04. Rate of pts with normal chest CT at 12 month was 17.9 vs 0%, P=0.10.
Fathi K M 2022 (31)	Iran	I/II	RCT	Severe COVID-19	Yes	15	MSC-derived secretome Standard care	5ml qd×5	15	Placebo Standard care	28-day survival rate was 57% vs 20%, P < 0.00 Pulmonary involvement improvement was 72.6 vs 28.7, P < 0.00
Karyana M 2022 (32)	Indonesia	I	RCT	Mild COVID-19	Yes	6	DW-MSCs Standard care	50-100 Once	3	Placebo Standard care	Treatment emergent AE was 3 vs 2 pts, P=0.595 All subjects recovered and discharged without complications.
Monsel A 2022 (33)	France	IIb	RCT	Severe COVID-19	Yes	21	UC-MSCs Standard care	1/kg qod×3	24	Placebo Standard care	Seventh-day survival rate 90.48 vs 87.50%, P=0.565; Twenty-eighth-day survival rate 76.19 vs 83.33%, P=410; ICU duration 15 vs 13 days, P=0.590; PaO2/FiO2-ratio change (day 0–7) 54.3 vs 25.3, P=0.77
Rebelatto C L K 2022 (34)	Brazil	I/II	RCT	Severe COVID-19	Yes	11	UC-MSCs Standard care	0.5/kg qod×3	6	Placebo Standard care	Fourteen-day survival rate was 81.81 vs 100%, P=0.404; Lung extension decreased at the fourth month.
Bowdish M E 2023 (35)	USA	III	RCT	Moderate-to-severe COVID-19	Yes	112	MSCs Standard care	2/kg biw×2	110	Placebo Standard care	Mortality rate at day 7 was 5.4 vs 4.5, RR=1.18(0.37, 3.75); Mortality rate at day 14 was 18.8 vs 21.8, RR=0.86(0.51, 1.45); Mortality rate at day 30 was 37.5 vs 42.7, RR=0.88(0.64, 1.21); Mortality risk at 12 month was similar, HR= 0.91(0.63, 1.30).

RCT, randomized control trial; BM, bone marrow; UC, umbilical cord; MB, menstrual blood; PL, placenta.

### 3.3 Drawbacks and risk of MSCs

The most controversial drawback is its uncertain effect on tumor generation. Some laboratory studies indicate that MSCs might promote cancer progression and therapeutic resistance. MSCs may secrete prostaglandin E2 (PGE2), IFN-γ, IL-4, TGF-β1, and VEGF to inhibit anti-tumor immune response (98), stimulate angiogenesis (99), promote cancer cells metastasis (100) and proliferation (101), and protect them from apoptosis (102), thereby promoting tumor growth. Furthermore, MSCs protect chronic myeloid leukemia cells and ovarian cancer cells against anti-tumor drugs by downregulation of caspase 3 *via* CXCL12/CXCR4 axis (103, 104). A similar phenomenon of drug resistance associated with MSCs was also observed in multiple myeloma and colorectal cancer *via* the production of CXCL13 (105) and IL-6 (106), respectively. Moreover, the multipotential of MSCs brings about concerns that MSCs themselves might be the potential cancer stem cells when MSCs are cultured for the long term (107).

Clinically, caution needs to be taken regarding some adverse events (AEs) of MSCs. A meta-analysis summarized the AEs of MSCs application over the past 15 years. The most frequent major AEs were fever and administration site conditions, and meaningfully common minor AEs were constipation, fatigue, and insomnia (108). No increasing risk of tumor generation had been observed (109). However, given the ethics principle, MSCs’ risk of promoting tumor relapse is still unclear because patients with cancer have been excluded from almost all clinical trials.

## 4 Conclusion

This is a rare case, with lung cancer and radiation pneumonitis, who suffered from severe infection and was successfully benefit from MSCs infusion. MSCs perhaps reverse the clinical outcomes by immunomodulation and tissue repair. Our case fills the gap of MSCs application for patients with carcinoma accompanied with severe pneumonia. MSCs may be an option for a refractory case in such a period without sufficient and effective drugs.

## Data availability statement

The original contributions presented in the study are included in the article/supplementary material. Further inquiries can be directed to the corresponding author.

## Ethics statement

The studies involving humans were approved by Ethics Committee of Yantai Yuhuangding Hospital. The studies were conducted in accordance with the local legislation and institutional requirements. The participants provided their written informed consent to participate in this study. Written informed consent was obtained from the individual(s) for the publication of any potentially identifiable images or data included in this article.

## Author contributions

XH: Investigation, Writing – original draft. XT: Investigation, Writing – original draft. XX: Investigation, Writing – original draft. TJ: Conceptualization, Writing – review & editing. YX: Supervision, Validation, Writing – review & editing. ZL: Conceptualization, Supervision, Validation, Writing – review & editing.
